# A maize seed variety identification method based on improving deep residual convolutional network

**DOI:** 10.3389/fpls.2024.1382715

**Published:** 2024-05-13

**Authors:** Jian Li, Fan Xu, Shaozhong Song, Ji Qi

**Affiliations:** ^1^College of Information Technology, Jilin Agricultural University, Changchun, China; ^2^College of Information Technology, Jilin Bioinformatics Research Center, Changchun, China; ^3^School of Data Science and Artificial Intelligence, Jilin Engineering Normal University, Changchun, China; ^4^College of Engineering Technical, Jilin Agricultural University, Changchun, China

**Keywords:** artificial intelligence, computer vision, corn seeds, variety identification, ResNet model

## Abstract

Seed quality and safety are related to national food security, and seed variety purity is an essential indicator in seed quality detection. This study established a maize seed dataset comprising 5877 images of six different types and proposed a maize seed recognition model based on an improved ResNet50 framework. Firstly, we introduced the ResStage structure in the early stage of the original model, which facilitated the network’s learning process and enabled more efficient information propagation across the network layers. Meanwhile, in the later residual blocks of the model, we introduced both the efficient channel attention (ECA) mechanism and depthwise separable (DS) convolution, which reduced the model’s parameter cost and enabled the capturing of more precise and detailed features. Finally, a Swish-PReLU mixed activation function was introduced globally to improve the overall predictive power of the model. The results showed that our model achieved an impressive accuracy of 91.23% in corn seed classification, surpassing other related models. Compared with the original model, our model improved the accuracy by 7.07%, reduced the loss value by 0.19, and decreased the number of parameters by 40%. The research suggested that this method can efficiently classify corn seeds, holding significant value in seed variety identification.

## 1 Introduction

Corn is the most widely grown cereal crop worldwide and is extensively used in food processing and as a primary component of animal feed (Shafinas et al., 2022). Seed purity refers to the degree of consistency in typical characteristics, directly impacting the yield and quality of corn. During seed harvesting and storage, impurities may inadvertently infiltrate average seeds, leading to economic losses in agricultural production and processing. During seed sales, some individuals or companies exploit inferior maize varieties to impersonate superior ones, aiming to make excessive profits (Sun and Zou, 2022). This erroneous behavior may damage investors’ interests and disrupt the seed market (Park et al., 2016). Therefore, an urgent need is to explore a non-destructive and efficient identification method for screening and grading maize seeds before they are marketed to ensure agricultural production, quality control, and market regulation (Tenaillon and Charcosset, 2011).

Traditional methods for seed purity identification include morphological inspection, field planting inspection, chemical identification, and electrophoresis technology (Ye-Yun et al., 2005; Sundaram et al., 2008; Pallavi et al., 2011; Satturu et al., 2018). However, these methods generally take a long time, require professional personnel and specialized equipment, and are often subject to the subjective experience of the testers. Additionally, the identification process may damage the samples. Hence, there is a need to develop a rapid, accurate, and non-destructive classification method for maize seed identification.

Deep learning has emerged as a critical research focus across various domains, particularly in the realm of computer vision. Integrating deep learning techniques with image processing has found widespread applications in seed classification and identification (Javanmardi et al., 2021; Tu et al., 2021). For instance, Zhu (Zhu et al., 2019) developed a self-designed Convolutional Neural Network (CNN) to classify seven varieties of cotton seeds, achieving an accuracy rate exceeding 80%—outperforming residual networks and other traditional models. Similarly, Rybacki (Rybacki et al., 2023) constructed a CNN with a fixed architecture comprising five alternating layers of Conv2D, MaxPooling2D, and Dropout. This model successfully identified seeds from three winter rapeseed varieties, attaining the highest validation accuracy of 85.6%. Atlanta (Altuntaş et al., 2019) employed a transfer learning approach using CNN to automatically differentiate between haploid and diploid corn kernels, achieving accuracy rates of over 90% across all models. In another study, Zhang (Zhang et al., 2020) proposed a deep learning model that combines near-infrared hyperspectral imaging (NIR-HSI) to determine the variety of coated maize seeds. Spectral reflectance values were extracted to train both CNN and Long Short-Term Memory (LSTM) models. The test results demonstrate that all models achieved classification accuracies exceeding 90%. Ma (Ma et al., 2020) integrated NIR-HSI and CNN deep learning techniques to differentiate between viable and non-viable seeds, achieving a seed detection rate of 90% in the process. Zhang (Zhang et al., 2021) investigated the feasibility of combining hyperspectral imaging (HSI) with deep CNN for classifying four varieties of maize seeds. The study showed that the classification performance of the deep CNN model was generally the highest among all varieties, with a validation accuracy of 93.3%. Yu (Yu et al., 2021) utilized HSI (948.17-1649.20nm) combined with CNN technology to identify 18 species of hybrid okra seeds, achieving a recognition rate of 93.79%. This demonstrates the reliable advantage of CNN models in achieving high accuracy and stability. Wang (Wang and Song, 2023) utilized hyperspectral imaging technology combined with deep learning methods to identify various varieties of sweet corn seeds. The results indicated that the deep learning model achieved a classification accuracy of over 95% on both the training and testing datasets. Bi (Bi et al., 2022) improved the Swin Transformer model and applied transfer learning to achieve high-precision classification and recognition of corn seed images, with an average accuracy of 96.53%. Xing (Xing et al., 2023) proposed a network model called GC_DRNet, incorporating the concept of dense networks and achieving an accuracy of 96.98% on a wheat seed dataset. Deep learning algorithms are gradually becoming optimal for establishing lossless detection models (Zhou et al., 2020; Zhang et al., 2023).

According to the studies above, efficient identification of seed varieties is challenging due to similar appearance, genetic diversity, and growth environment. Therefore, combining neural networks and hyperspectral data has been predominantly relied upon to recognize seed varieties effectively. Although this approach outperforms using convolutional neural networks alone for recognition, acquiring hyperspectral data is not easily accessible, and the processing involved is complex. In order to address the limitations above, this study is based on a pure image dataset of corn seeds. By improving the classical ResNet50 model, a new convolutional neural network model for corn seed identification is proposed. The main contributions and novelties of this work are listed as follows.

1. We introduced the IResStage structure in the early stages of ResNet50, enhancing the residual blocks to improve the model’s feature extraction and network representation capabilities. This enables it to capture and convey image features more effectively.
2. In the later stages of the network, we incorporated the ECA module and depthwise separable convolution. The attention mechanism strengthens the focus on channel information, while the use of depthwise separable convolution aims to reduce time costs, further enhancing the model’s ability to capture more precise and detailed features, thereby increasing the efficiency of model recognition.
3. We introduced a global hybrid activation function by combining different activation functions, enhancing the model’s generalization ability and accuracy during the prediction phase, allowing it to process input data better and make more accurate predictions.

The following of this article was organized as the section “Materials and Methods” described the details of the datasets and the overview of the methods, the experimental results were described and discussed in the section “Results and Discussions”, and the section “Conclusions” was the concluding remarks.

## 2 Materials and methods

### 2.1 Image acquisition and preprocessing

#### 2.1.1 Data source and acquisition

**Figure 1:**
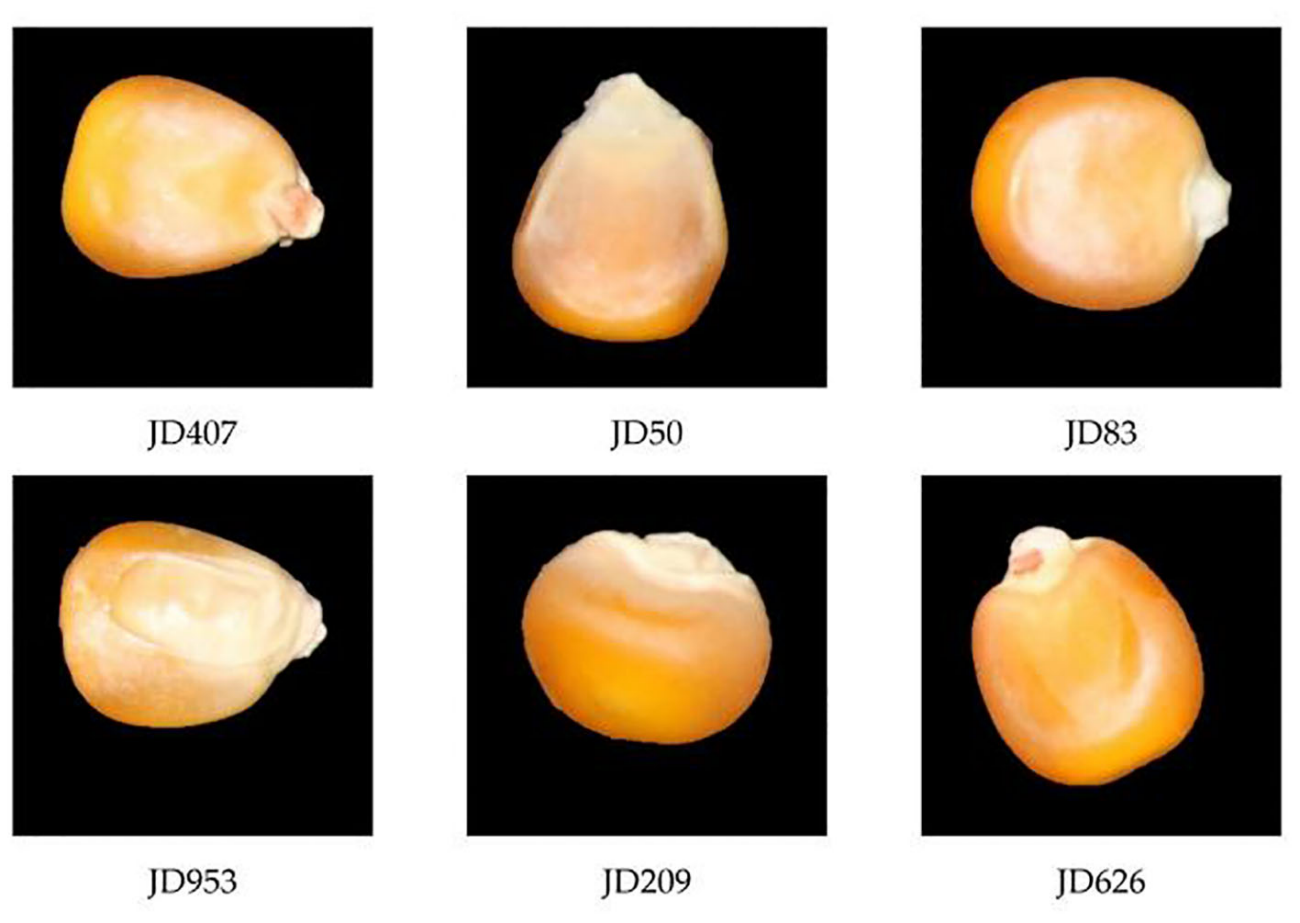
Image of maize seed varieties.

The six different varieties of corn seeds, including JD407, JD50, JD83, JD953, JD209, and JD626, were provided by the Corn Institute of Jilin Academy of Agricultural Sciences in Jilin Province. These seeds were photographed using a Canon 70D camera. The high-definition color images are shown in **Figure 1**. During sampling, experts selected and certified the seeds and manually screened them to select whole, uniformly shaped seeds as experimental samples while removing impurities and dust. Subsequently, image acquisition work was carried out. All samples appeared normal, displaying a neat exterior without any visible damage. Approximately 900 to 1000 samples were randomly selected from each variety for imaging and stored in sealed plastic packaging at room temperature (20 ± 1°C). This sample size is because deep learning networks require a large number of samples for proper training (Wen, 2020).

#### 2.1.2 Image preprocessing and segmentation

**Figure 2:**
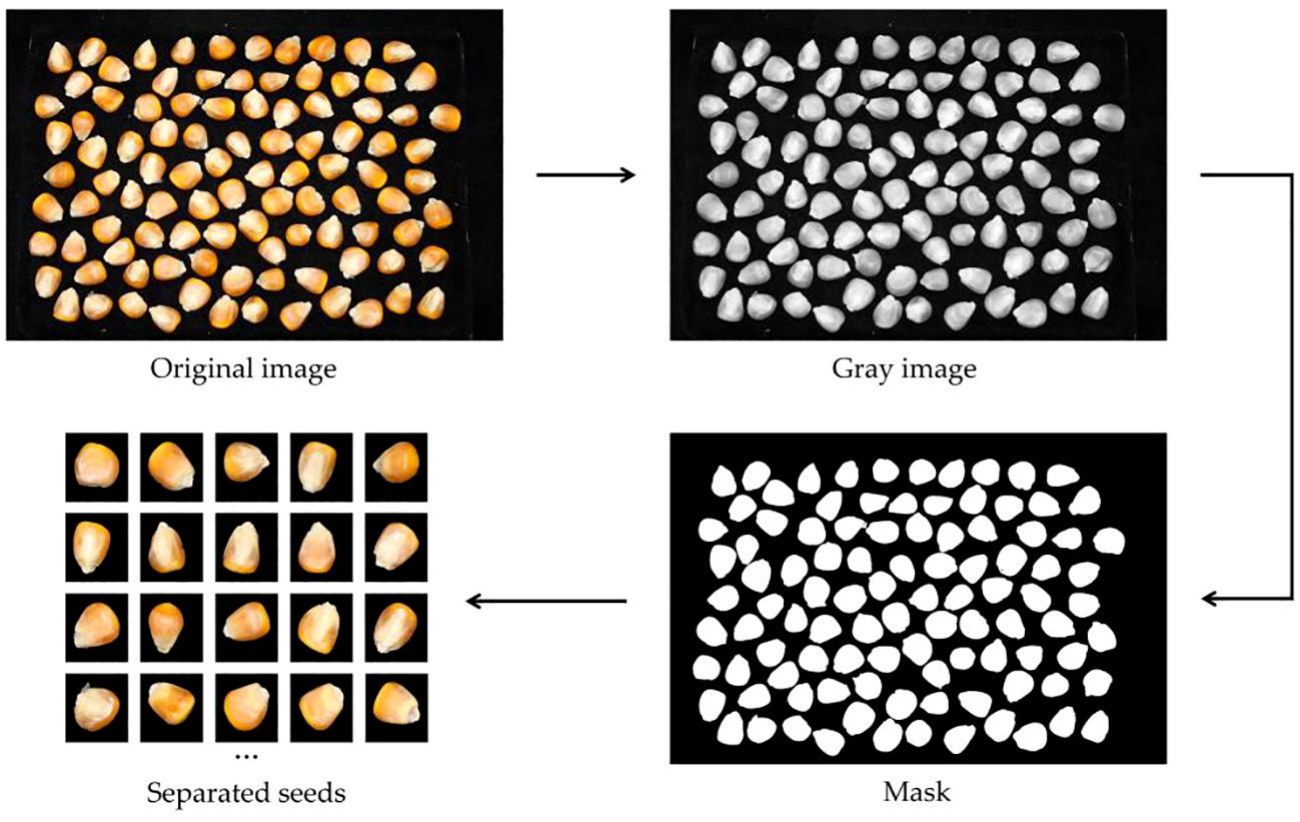
Corn seed image cutting processing.

The research on maize variety identification focuses on authenticating and ensuring the purity of maize seeds. Seed purity comprises the authenticity of individual seeds. A single-seed identification method is employed to identify the variety of seeds, requiring the segmentation of images containing multiple maize seeds. Firstly, the image is converted to a grayscale image to facilitate the removal of color information and to highlight brightness-related features. Subsequently, automatic global thresholding and morphological filtering are then applied to obtain a binary image, simplifying the image and extracting the contours of the targets. Finally, morphological filtering of the binary image is used to score the mask of the maize seed region. This is then used to partition it into individual maize seeds of size 224*224, resulting in a total of 5877 original images. The image cutting process is shown in **Figure 2**.

Each model underwent training using a 5-fold cross-validation method to address the many uncertainties in the experiments. 80% of the dataset is randomly selected as the training set and 20% as the test set. Due to the limited sample size and to ensure the generalization ability of the model, the validation set is also used as the test set to evaluate the results. The dataset comprised 4703 images for training and 1174 images for validation, as shown in **Table 1**. Therefore, the final experimental results in this paper were based on the average of the results of five experiments.

**Table 1:** Dataset partition.

Seed Variety	Training Set	Validation Set	Totals
JD407	781	195	976
JD50	781	195	976
JD83	781	195	976
JD953	768	192	960
JD209	799	199	998
JD626	793	198	991

### 2.2 Building the model

#### 2.2.1 ResNet50 model

**Figure 3:**
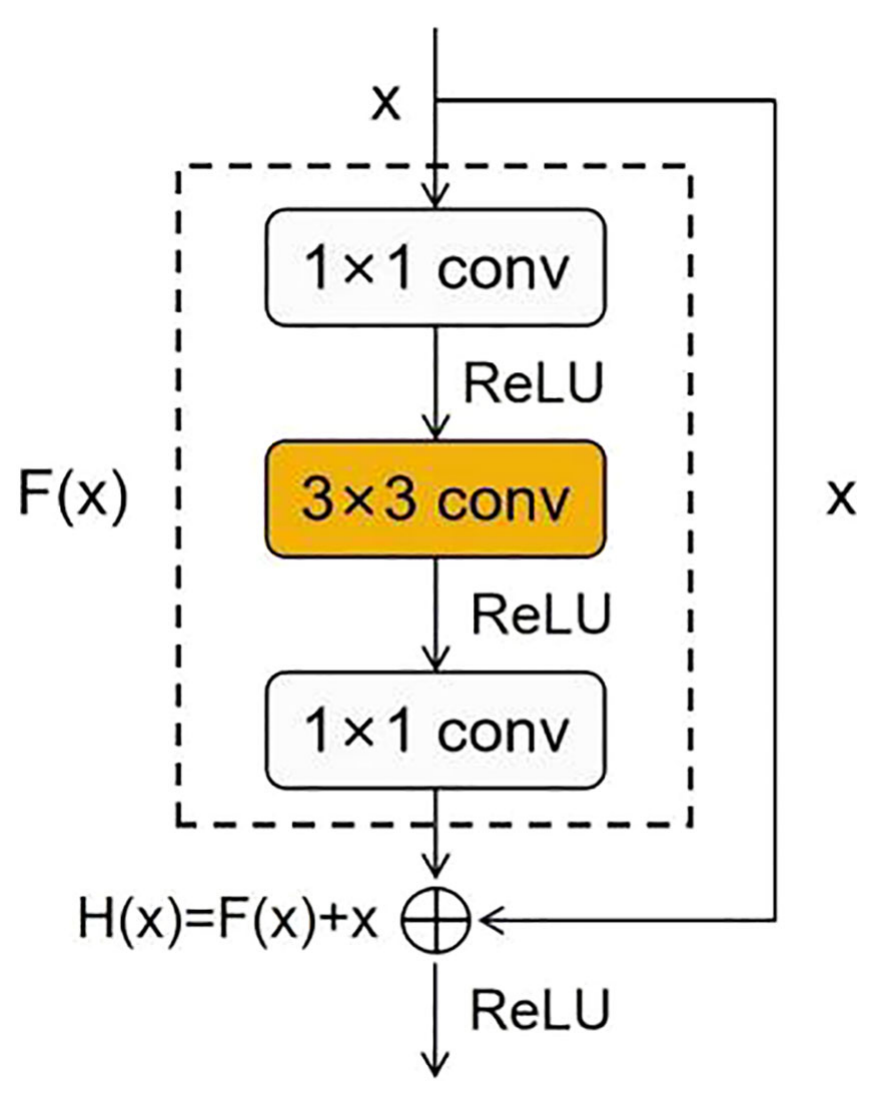
Residual block.

ResNet is a deep neural network proposed by He et al (He et al., 2016). Due to its deeper network structure, unique residual connection design, and higher parameter efficiency, it can learn complex features, alleviate the vanishing gradient problem, and exhibit good generalization ability while maintaining a relatively fast inference speed. Due to these advantages, ResNet50 has become an ideal model for maize seed recognition, effectively extracting useful information from images for classification purposes. The residual block is an essential structure of ResNet50, which addresses the vanishing gradient and exploding gradient problems in deep neural networks by introducing skip connections and identity mapping. The residual block consists of two primary operations: the main path and the shortcut connection. The main path comprises a series of convolutional, normalization, and non-linear activation layers, which extract high-level representations of the input features. The shortcut connection is a simple mapping that achieves cross-layer information propagation by directly adding the input to the output of the main path. The structure of the residual block is shown in **Figure 3**.

#### 2.2.2 ResStage structure

**Figure 4:**
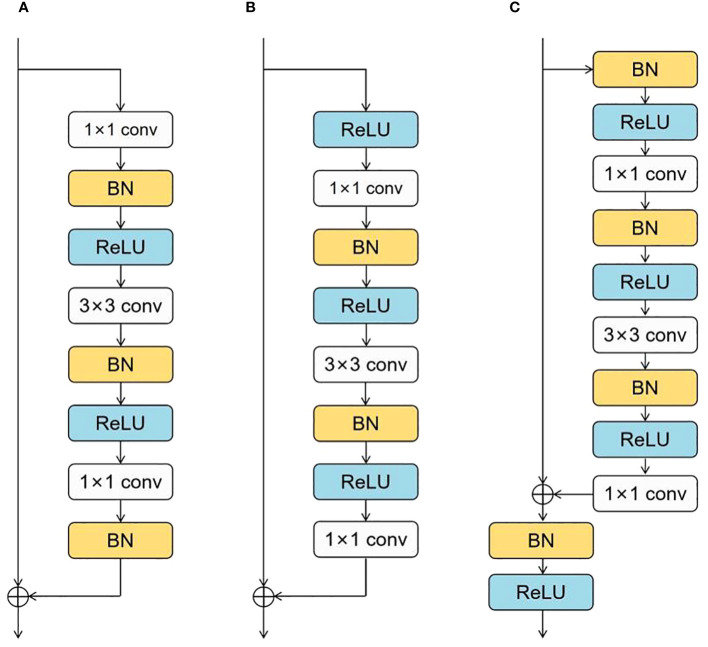
ResStage structure: **(A)** Start ResBlock, **(B)** Middle ResBlock, **(C)** End ResBlock.

To facilitate the network’s learning process, we need to provide better pathways for information propagation across network layers. C. Duta et al. proposed a simple, practical, stage-based CNN module called the ResStage structure (Duta et al., 2020), as shown in **Figure 4**. The ResStage structure has modified the arrangement of components, dividing each central stage into three parts: a Start ResBlock, a Middle ResBlock, and an End ResBlock. The Start ResBlock includes a BN layer after the last conv operation, preparing for element-wise addition through normalization. The End ResBlock is completed by BN and ReLU operations, preparing for a stable transition into the next stage. The module aims to achieve efficient information flow while maintaining controlled signal propagation through learning in these three stages.

In the original residual block, the number of ReLU units on the main propagation path is directly proportional to the network depth. In contrast, ResStage contains a fixed number of ReLU units on the main path, facilitating forward and backward information propagation. In the main stage, there are only four ReLU units along the main information propagation path, and they are not affected by changes in depth. This design enables the network to prevent signal obstruction as information passes through multiple layers, enhancing information extraction and learning capability. The complexity of maize seed morphology may challenge traditional feature extraction methods in capturing all essential features. The ResStage structure effectively reduces information loss, extracts more comprehensive feature information, prevents model gradient vanishing, and reduces hyperparameter demand.

#### 2.2.3 Improved residual block

**Figure 5:**
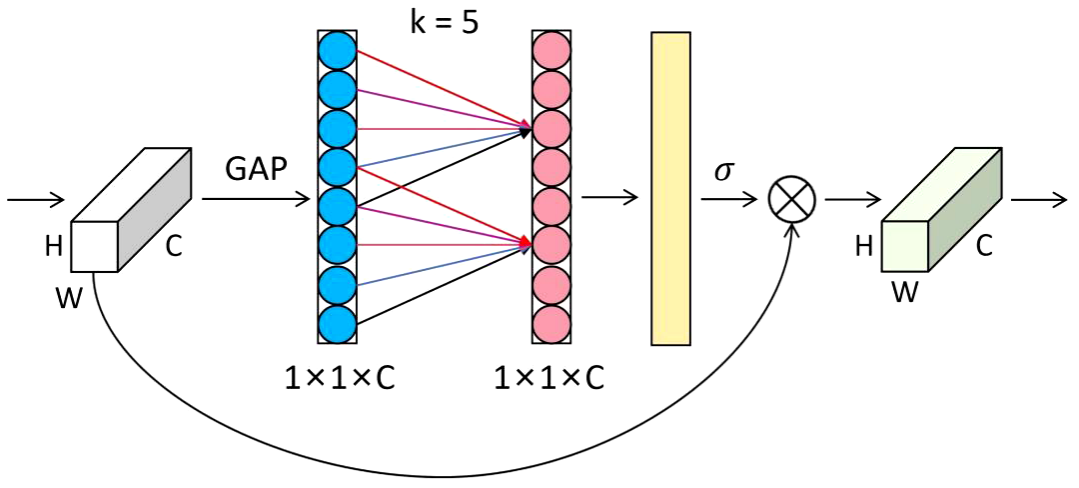
Structure of the efficient channel attention module.

The attention mechanism plays a crucial role in deep learning, effectively and accurately filtering out valuable information from a large amount of data. This is highly beneficial for various image-processing tasks (Mi et al., 2020; Zang et al., 2022; Feng et al., 2024). Therefore, in this study, we introduced an attention mechanism called ECA (Efficient Channel Attention) after the first convolution of the subsequent residual block (Wang et al., 2020). The ECA module can adaptively adjust the weights of channel features, allowing the network to focus on essential features better. Most maize seeds have similar shapes and delicate textures, affecting recognition after downsampling and making it difficult to extract detailed features from the network. The ECA module helps improve the discriminative ability of features and suppress unimportant features, thereby reducing the risk of overfitting. Ultimately, this enhances feature representation and improves the model’s generalization ability without significantly increasing computational costs. The structure of the ECA module is shown in **Figure 5**.

The forward process of the ECA module is as follows: first, the input feature map with a size of H×W×C undergoes global average pooling to obtain feature information. Then, new weight values ω are generated through a one-dimensional convolution of size k and a sigmoid activation function, completing inter-channel information interaction, as shown in Equation (1).


(1) $$\omega =\sigma \left(\text{C}1{\text{D}}_{\text{k}}\left(\text{y}\right)\right)$$

where $\text{C}1{\text{D}}_{\text{k}}$ represents a one-dimensional convolution with a kernel size of k, and σ is the sigmoid activation function. The number of channels C is proportional to the one-dimensional convolution with kernel k, as shown in Equation (2).


(2) $$\text{C}=2^{\left(\text{γ*k}-\text{b}\right)}$$

Thus, we can obtain the final kernel size k, as shown in Equation (3).


(3) $$\text{k}={\left|\;\frac{{\text{log}}_{2}\left(\text{C}\right)}{\text{γ}}+\frac{\text{b}}{\text{γ}}\;\right|}_{\text{odd}}$$

where t is the nearest odd number to ${\left|\text{t}\right|}_{\text{odd}}$ , γ is 2, and b is 1.

**Figure 6:**
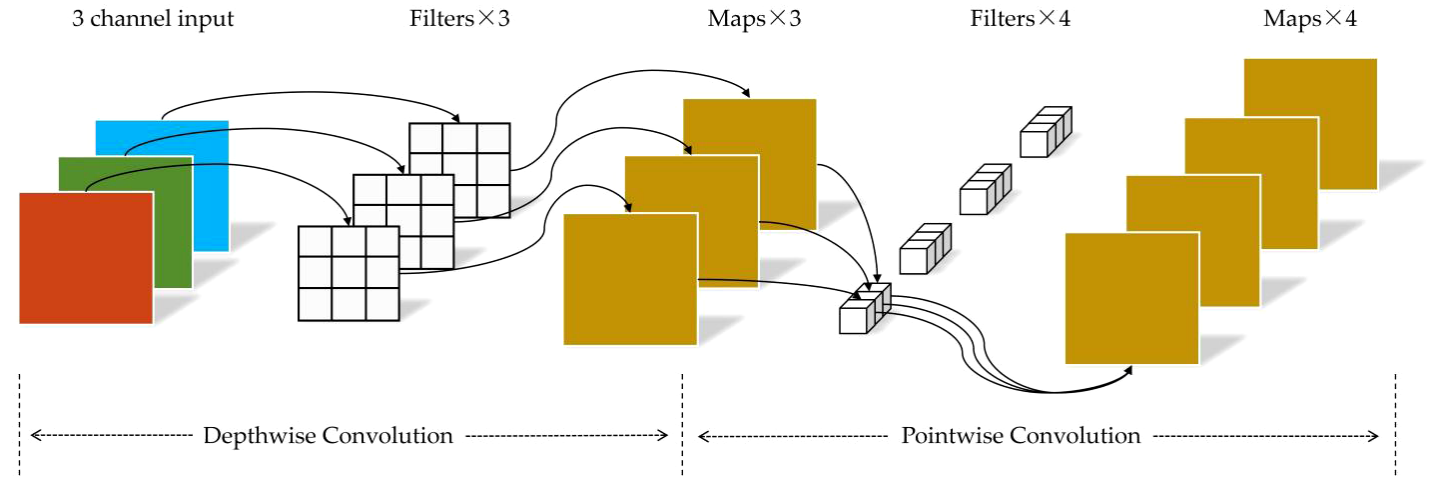
Structure of the depthwise separable convolution.

In addition, to reduce the computational cost and time consumption of the network model, we incorporate depthwise separable convolution (Chollet, 2017) into the subsequent residual blocks of the maize seed recognition model. Depthwise separable convolution consists of two sub-layers: depthwise convolution and pointwise convolution, as illustrated in **Figure 6**. In the first stage of depthwise convolution, convolution operations are performed individually on each channel. In the second stage of pointwise convolution, the number of channels is adjusted to match a predefined output channel number. Unlike conventional convolution, where each kernel operates on the entire input volume, each kernel is responsible for a single channel in depthwise convolution. For example, in a three-channel color image, the first stage of depthwise convolution performs a two-dimensional convolution operation for each channel independently, resulting in three feature maps. Subsequently, the pointwise convolution process is akin to traditional convolution, as it entails a weighted combination of the preceding feature maps along the channel dimension to produce new feature maps.

Depthwise convolution utilizes a single convolution kernel to perform channel-wise convolutions on input channels, effectively reducing computational complexity and accelerating forward and backward propagation, lowering computation and storage costs. Furthermore, depthwise separable convolution combines information from different channels through pointwise convolution, thus preserving a specific feature extraction capability.

**Figure 7:**
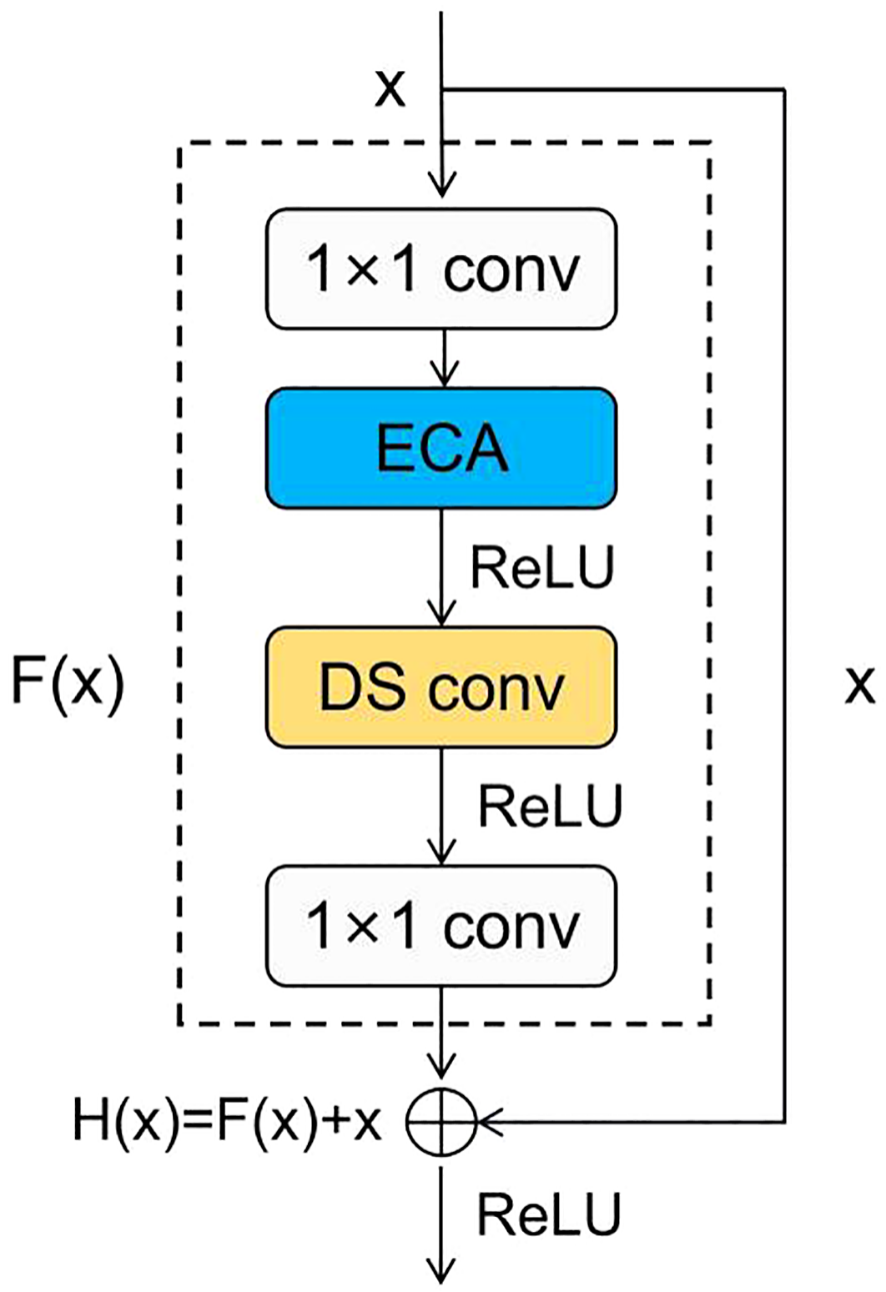
Structure of the improved residual block.

In conclusion, we incorporated the ECA module into the subsequent residual blocks to efficiently recognize maize seeds and replaced the second convolution with a depthwise separable convolution. This enables us to reduce the model parameter count while enhancing the model’s overall performance. The improved residual block is shown in the **Figure 7**.

#### 2.2.4 Using mixed activation functions

**Figure 8:**
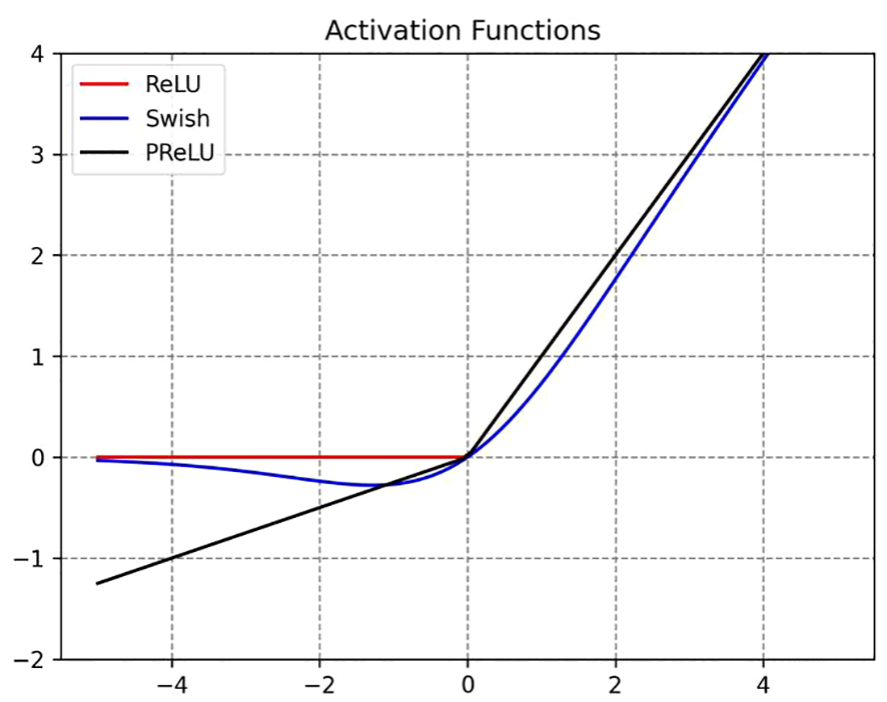
ReLU, Swish and PReLU activation function curves.

The activation function is a non-linear function used to increase the non-linearity of the network model between the output of upper-layer nodes and the input of lower-layer nodes in a multi-layer neural network (Ohn and Kim, 2019). For a specific training model, selecting an appropriate activation function can effectively improve the neural network’s performance (Apicella et al., 2021). In order to maximize the expressive power of the model, this paper selects the Swish and PReLU activation functions to replace the original ReLU function at different positions. The corresponding image is shown in **Figure 8**.

ReLU is the most commonly used activation function, which effectively alleviates the gradient vanishing problem in deep neural networks. Its proposal has led to significant advances in the field of deep learning (Wang et al., 2018). The expression is defined as shown in Equation (4):


(4) $$\text{ReLU}\left(\text{x}\right)\;=\{\begin{array}{c} \;\;0,\;\;\;x\;\leq \;0\\ \;\;x,\;\;\;x\;>\;0 \end{array}$$

where x is the input. When the input value is less than or equal to 0, the gradient of ReLU is 0, which means that the neuron becomes “dead” and cannot update its weights, resulting in information loss. Therefore, the PReLU activation function was proposed to address the issues of the ReLU function (He et al., 2015). The expression is defined as shown in Equation (5):


(5) $$\text{PReLU}\left(\text{x}\right)\;=\{\begin{array}{c} \;\;\;\;0,\;\;\;\text{x}\;\leq \;0\\ \;\;\text{αx},\;\;\;\text{x}\;>\;0 \end{array}$$

where x is the input, and α is a learnable parameter. PReLU is an improvement over LReLU, as it can adaptively learn parameters from the data, offering the advantages of fast convergence and low error rates. Additionally, PReLU can be used for backpropagation training and can be jointly optimized with other layers.

Swish is a novel composite activation function (Ramachandran et al., 2017), and its expression is defined as shown in Equation (6):


(6) f(x)=x·sigmoid(x)

where x is the input. The Swish activation function possesses the characteristics of having no upper bound, a lower bound, smoothness, and non-monotonicity, which can alleviate the gradient vanishing problem. Furthermore, its performance in deep models surpasses that of the ReLU activation function.

The PReLU and Swish activation functions can, to some extent, address the drawbacks of the ReLU activation function. Therefore, in this study, a combination of these two activation functions is employed to replace the ReLU function at different positions, aiming to enhance the model’s predictive capability for maize seed classification.

#### 2.2.5 Proposed model

**Figure 9:**
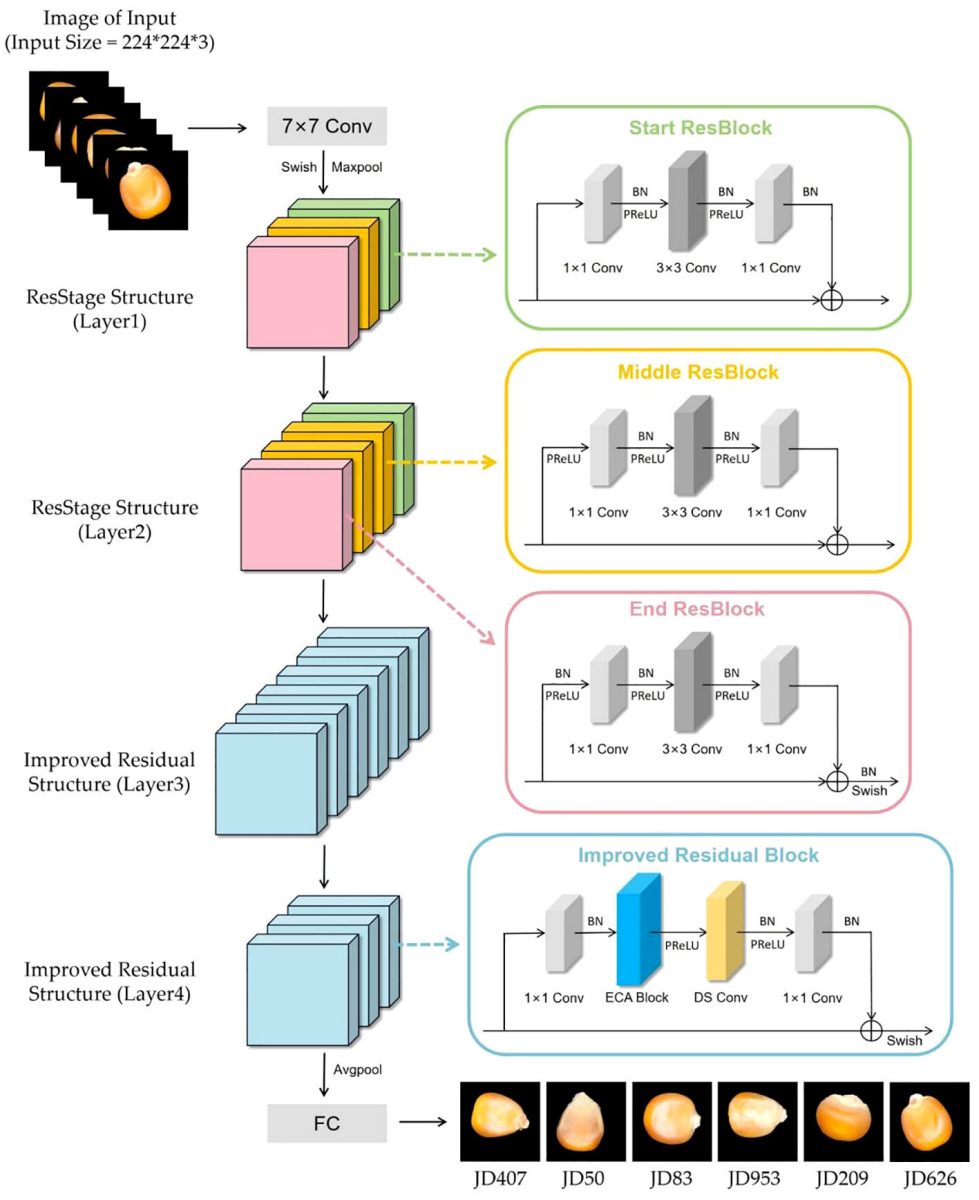
The improved model.

To minimize information loss during the recognition process of corn seeds, we introduced the ResStage structure in the early stages of our model. This structure optimizes the positioning of BN layers and the ReLU activation function, effectively mitigating the negative impact of non-linear activations on information propagation. These adjustments significantly enhance feature extraction and information propagation capabilities. Furthermore, we enhanced the residual structure in later stages by incorporating the ECA module and depthwise separable convolution into each residual block. This enhancement fosters effective feature interaction while reducing computational costs, thus improving recognition capabilities. Lastly, we globally integrated a mixed activation function into the model. We replaced the activation functions after skipping connections in the End ResBlock and improved residual blocks, as well as the initial activation function in the network input layer, with the Swish activation function. Additionally, in all other positions, we replaced the activation function with the PReLU activation function to enhance the overall predictive capacity of the model. The improved model is shown in the **Figure 9**.

## 3 Results and discussions

### 3.1 Experimental setup

The configuration environment for this antler classification experiment is: processor: Xeon 5220R, graphics card: NVIDIA TESLA T4, operating system: windows10, Python3.8.16 based Pytorch1.13.1 deep learning framework built on Python3.8.16 programming language, software configuration installed as Anaconda3-2021.11- windows version. The specific parameter settings in the experiment are shown in **Table 2**.

**Table 2:** Training hyperparameter information.

Parameter	Value or Name	Description
Training epochs	150	The complete number of times the entire training dataset goes through forward and backward propagation through the neural network
Batch size	32	The number of samples used to calculate gradients and update model parameters in a single training iteration
Learning rate	0.001	A hyperparameter that controls the magnitude of model weight adjustments
Momentum	0.9	An optimization technique used to accelerate convergence and reduce fluctuations during parameter updates
Weight decay	0.0005	A regularization technique used to suppress model complexity and prevent overfitting
Optimizer	SGD	An optimization method used for updating and calculating neural network model parameters to reduce the value of the loss function

To select the optimal learning rate, comparative experiments were conducted with the learning rate set to 0.01, 0.001, and 0.0001, respectively, to determine the best parameters. The test results are shown in **Table 3**. The experimental results indicate that when the learning rate was 0.001, the original model achieved the highest recognition accuracy in the test set, at 84.16%, higher than the models with other parameters. Therefore, it was confirmed that a learning rate of 0.001 is the training parameter.

**Table 3:** Performance comparison results of different learning rate.

Learning rate	Model	Accuracy	Loss
0.01	ResNet50	82.62%	0.5255
0.001	ResNet50	84.16%	0.4649
0.0001	ResNet50	77.34%	0.6478

### 3.2 Comparison experiments of different models

**Figure 10:**
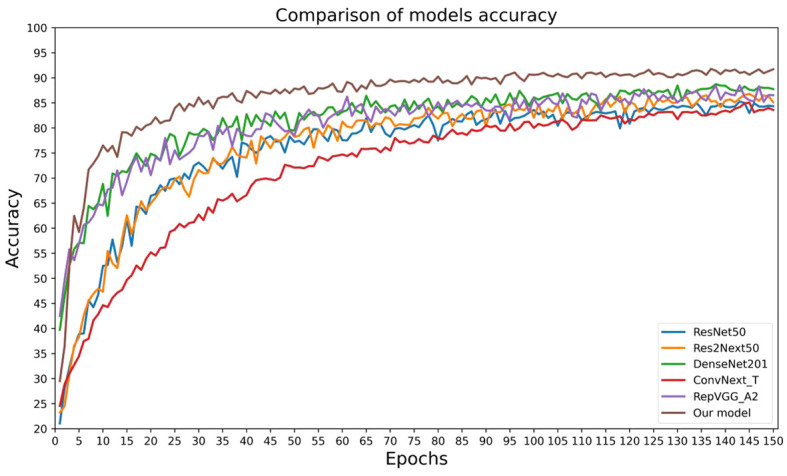
Results of the accuracy of different model comparison experiments.

**Figure 11:**
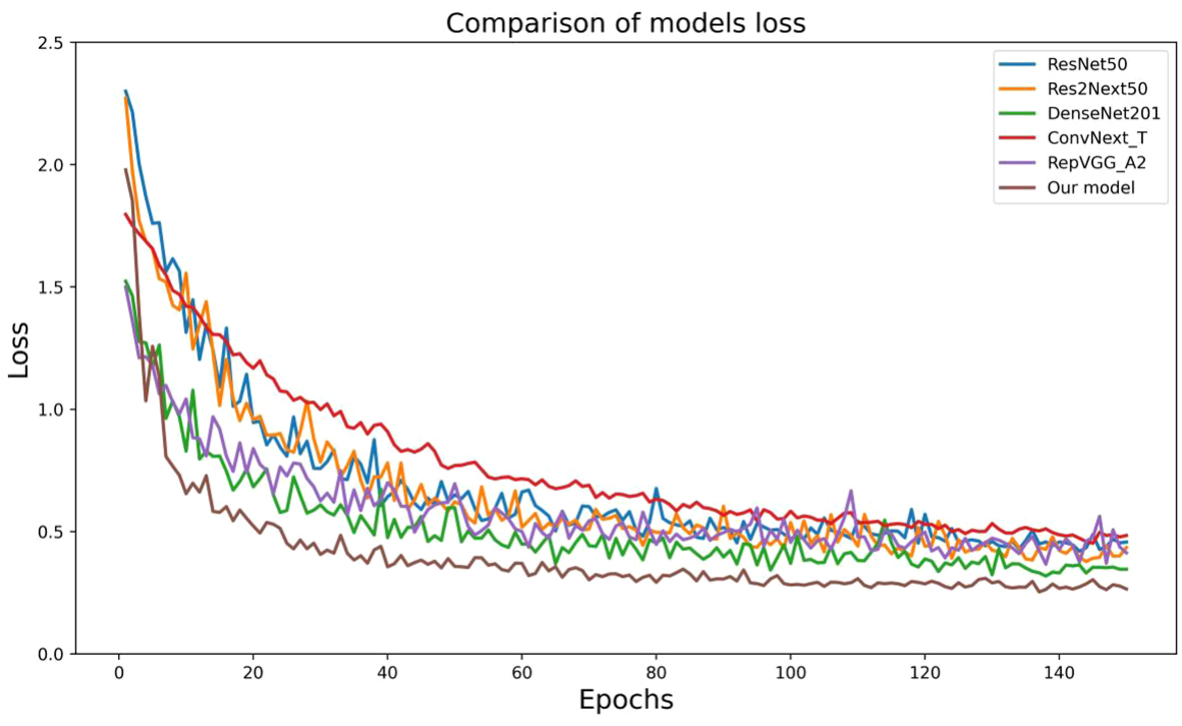
Results of the loss of different model comparison experiments.

In order to validate the effectiveness and advancement of the new network model, we used model accuracy, model loss, model parameters, model floating-point operations per second (FLOPs), and model training time per epoch as evaluation metrics for the model’s performance. We compared the new network model with five classic convolutional neural networks (ResNet50, Res2Next50, DenseNet201, ConvNext_T, and RepVgg_A2) to assess its performance. The results are shown in **Table 4**, **Figures 10**, **11**.

**Table 4:** Comparison experiments of different models.

Model	Accuracy	Loss	Params	FLOPs	Time/Epoch
ResNet50	84.16%	0.46	23.52 M	4.12 GMac	60s
Res2Next50	85.43%	0.43	22.63 M	4.21 GMac	104s
DenseNet201	87.47%	0.36	18.10 M	4.37 GMac	85s
ConvNext_T	83.25%	0.50	27.82 M	4.48 GMac	75s
RepVGG_A2	86.52%	0.44	26.81 M	5.70 GMac	54s
Our model	91.23%	0.27	14.12 M	3.20 GMac	57s

By analyzing the results of comparative experiments, this paper’s proposed corn seed classification model achieved the best accuracy of 91.23%. It also demonstrated the lowest loss value of 0.27, the lowest parameter count of 14.12 M, the lowest FLOPs value of 3.2 GMac, and a running time of only 57s per epoch. Compared to the original model, it showed an improvement of 7.07% in accuracy, a reduction of 0.19 in loss value, a 40% decrease in parameter count, a decrease of 0.92 GMac in FLOPs, and a 3s acceleration in running time per epoch. In comparison, other models exhibit slower recognition speed, lower accuracy, and weaker generalization ability when classifying corn seed image samples. These findings provide evidence for the superior performance of the proposed model in this paper, as it converges rapidly to find the optimal values. This proves the superior performance of the model, which converges quickly to find the best value.

### 3.3 Ablation experiments

To assess the impact of the ResStage structure, improved residual structure, and mixed activation functions on model performance, we conducted ablation experiments using ResNet50 as the base network. The results, as shown in **Table 5**, indicate that integrating these three modules enhances model performance, thereby improving its suitability for classifying maize seed varieties. Furthermore, the simultaneous integration of these modules further enhances model accuracy, providing more reliable and precise classification results for maize seed classification.

**Table 5:** Comparison of ResNet50 experimental models with different module combinations.

Num	ResNet50	ResStage Structure	Improved Residual Block	Activation Function	Accuracy	Params
1	√				84.16%	23.52 M
2	√	√			86.62%	23.52 M
3	√		√		87.99%	14.12 M
4	√			√	85.43%	23.52 M
5	√	√	√		90.11%	14.12 M
6	√	√		√	87.79%	23.52 M
7	√		√	√	89.16%	14.12 M
8	√	√	√	√	91.23%	14.12 M

#### 3.3.1 Effect of depthwise separable convolution on network model performance

This study replaced traditional convolution operations with depthwise separable convolutions, which embrace the concept of lightweight design. Compared to the original model, the accuracy improvement was only 0.6%. However, by restructuring the residual blocks while ensuring a slight increase in accuracy, there was a significant reduction in the number of model parameters. This change enhanced the model’s floating-point computation capabilities, ultimately leading to a practical improvement in the model’s training efficiency. The overall results of the model before and after the introduction of depthwise separable convolutions are shown in **Table 6**.

**Table 6:** Comparison results before and after adding depthwise separable convolution to the model.

Num	ResNet50	Other Modules	DS Conv	Accuracy	Params	FLOPs
1	√	√	None	90.63%	23.52 M	4.13 GMac
2	√	√	√	91.23%	14.12 M	3.20 GMac

#### 3.3.2 Effect of attentional mechanisms on network model performance

**Figure 12:**
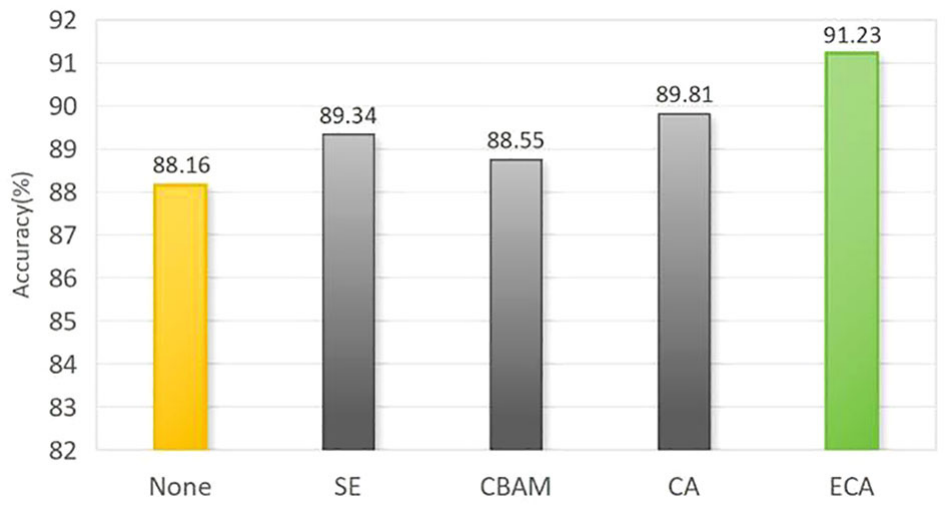
Recognition results comparison of different attention mechanism models.

Adding appropriate attention mechanisms in the network can enhance its ability to extract effective image features. In this experiment, we kept other factors constant and introduced different attention mechanisms into the proposed maize seed classification model for comparison. The results are shown in **Figure 12**; after introducing the Squeeze-and-Excitation(SE), Convolutional Block Attention Module(CBAM), Coordinate attention(CA), and ECA modules, the model’s accuracy increased by 1.18%, 0.59%, 1.65%, and 3.07%, respectively, compared to the original model. Among them, the ECA module has a more significant effect on improving network performance. This indicates that by efficiently and accurately calculating attention across channel dimensions, the ECA module can better capture the dependency between features, utilize contextual information, and suppress irrelevant noise, thereby achieving better performance in the task of maize seed recognition.

To intuitively analyze the effectiveness of the improved maize seed classification model, we utilized the visualization tool Grad-CAM (Selvaraju et al., 2016). Grad-CAM visualizes the image regions focused on by the model during prediction by calculating the gradients of the target class concerning the feature maps, multiplying these gradients with the feature maps to obtain weights, and ultimately generating a heatmap. The original images are displayed in the first row, while the second and third columns show the Grad-CAM mapping images before and after incorporating the ECA module. The color spectrum from red to blue indicates the degree of contribution.

**Figure 13:**
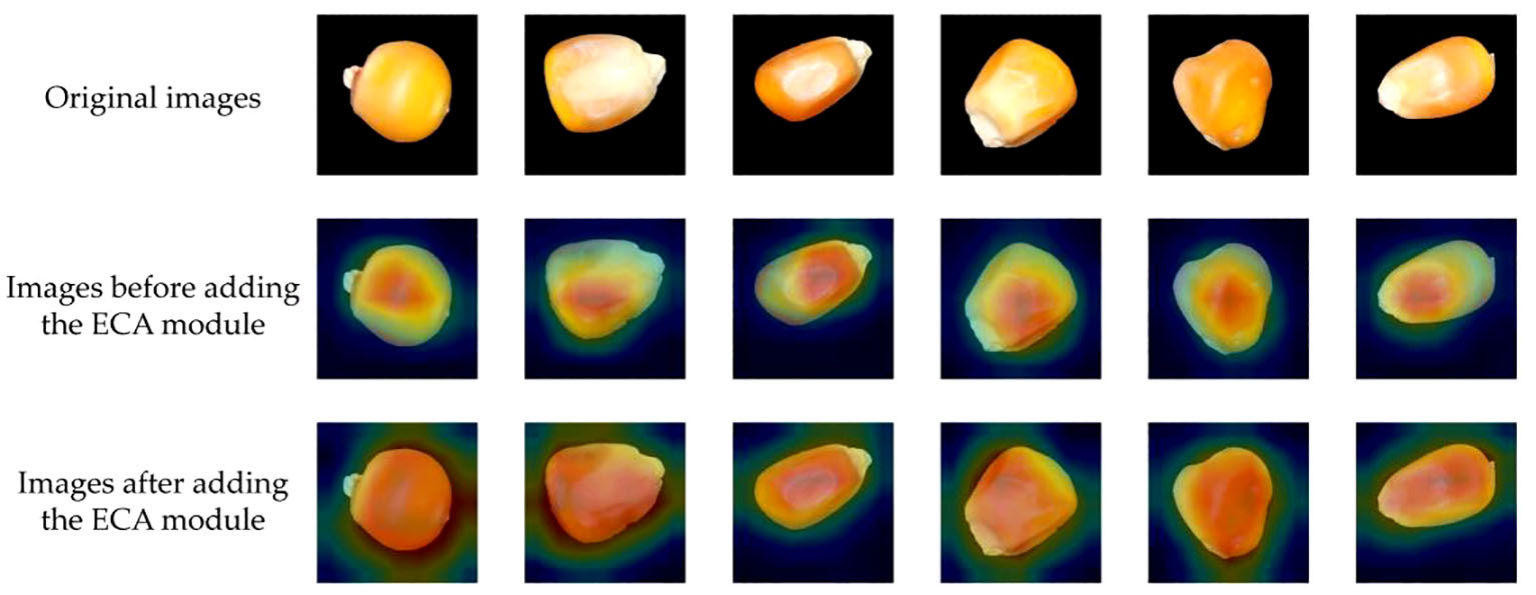
Visualization results of the new network thermal characteristic map before and after improvement.

The visualization of the experimental results is shown in **Figure 13**. Before the introduction of the attention mechanism, the model might have focused more on the local features of the seeds, possibly due to the model’s insufficient grasp of the global features of the entire image. Consequently, the heatmaps mainly concentrated on the local areas of the seeds, causing the model to prioritize certain local features while neglecting overall features during prediction. However, after incorporating the ECA module, the model’s attention to channel information increased, enhancing its ability to grasp global features. This enabled the model to better focus on the features of the entire seed, not just the local features, during prediction. Therefore, the ECA module has enhanced the feature extraction capability of the corn seed classification model, enabling it to locate valuable areas within the corn seed images more accurately.

#### 3.3.3 Effect of mixed activation function on network model performance

**Figure 14:**
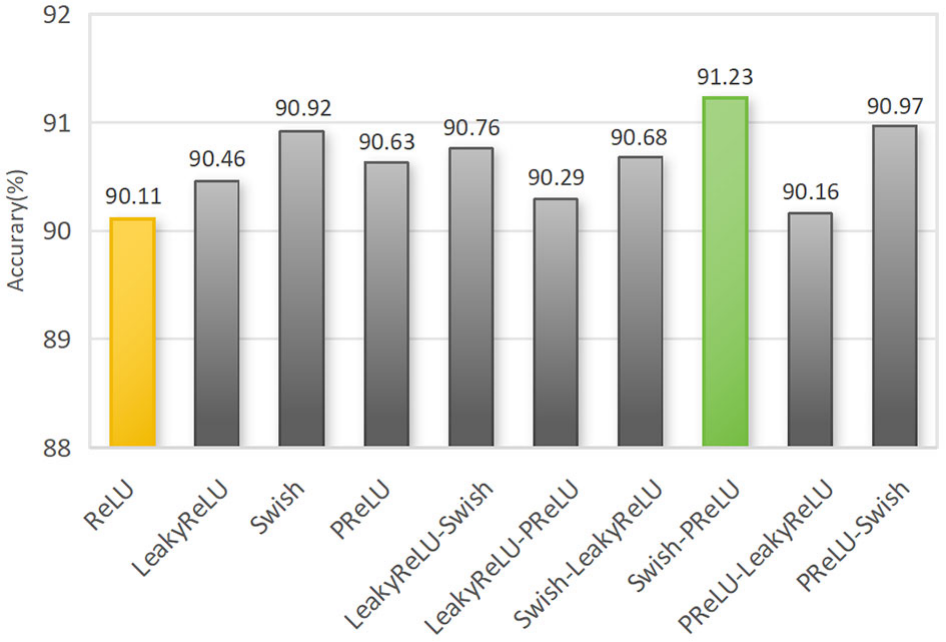
Recognition results comparison of for different combinations of activation functions. Note: A single name represents the global activation function. The activation function before the “-” symbol denotes activation 1, while the activation function after the “-” symbol denotes activation 2.

The choice of activation function is also crucial during the training process, as it significantly impacts the performance of the same model. We experimented with three activation functions (LeakyReLU, Swish, and PReLU) and the original ReLU activation function to improve the ResNet50 network architecture. We explored the impact of mixed activation functions on the performance of deep networks. We divided the overall activation functions into two categories. Activation 1 represents the activation function used after skipping connections in the End ResBlock and improved residual blocks, and the first activation function is in the network input layer. Activation 2 represents another activation function used in other positions. The results are shown in **Figure 14**.

The results indicate that compared to the original global use of the ReLU activation function, the accuracy improved by 1.12% when using the Swish-PReLU mixed activation function. It outperformed other global activation functions and combinations of mixed activation functions. The Swish activation function, with its non-zero mean within the input range, preserves more information and helps enhance the expressive power of the model. On the other hand, the PReLU activation function provides more detailed information to maximize inter-class differences, such as the texture, lines, and colors of corn seeds, enabling the extraction of detailed features that are challenging to capture. Using the Swish-PReLU mixed activation function, we can leverage the advantages of both functions to achieve better generalization performance and recognition results. This significantly improves the performance of the corn seed classification model.

### 3.4 Comparison of relevant indicators

This article also cites three metrics: precision, as seen in Equation 7 (Kosmopoulos et al., 2015), recall, as seen in Equation 8 (Zhu et al., 2010), and F1-score, as seen in Equation 9 (Hai et al., 2017), as evaluations of the model’s performance on different classes. Precision refers to the probability of a specific category being correctly predicted among all predicted results. Recall refers to the probability of a specific category being correctly predicted among all actual values. The F1 score is the harmonic mean of precision and recall.


(7) $$\text{Precision}=\frac{\text{TP}}{\text{TP}+\text{FP}}$$

(8) $$\text{Recall}=\frac{\text{TP}}{\text{TP}+\text{FN}}$$

(9) $$\text{F}1=2\times \frac{\left(\text{Precision }\times \text{ Recall}\right)}{\left(\text{Precision }+\text{Recall}\right)}$$

where TP refers to the correctly classified positive samples, FP refers to the negative samples mistakenly classified as positive, TN refers to the correctly classified negative samples, and FN refers to the positive samples mistakenly classified as negative.

The results from the **Table 7** demonstrate that the improved model, when compared to the original model, has enhanced various indicators for all six types of corn seeds. The Precision for each category of corn seeds has increased by 5.7%, 4.8%, 3.5%, 13.2%, 8.3%, and 6.7% respectively. The Recall has seen improvements of 10.3%, 12.3%, 1%, 4.6%, 2.6%, and 11.6% respectively. Furthermore, the F1 scores have shown improvements of 0.08, 0.085, 0.022, 0.089, 0.056, and 0.093 respectively. These findings indicate that the improved network exhibits better recognition performance in the classification of corn seed images.

**Table 7:** Comparison of model recognition performance evaluation metrics.

Label	Seed Category	Precision		Recall		F1-score	
		Before	After	Before	After	Before	After
1	JD407	86.8%	92.5%	84.6%	94.9%	0.857	0.937
2	JD50	82.2%	87.0%	80.5%	92.8%	0.813	0.898
3	JD83	91.7%	95.2%	90.8%	91.8%	0.912	0.935
4	JD953	79.7%	92.9%	83.9%	88.5%	0.817	0.906
5	JD209	80.0%	88.3%	88.4%	91.0%	0.84	0.896
6	JD626	85.4%	92.1%	76.8%	88.4%	0.809	0.902

**Figure 15:**
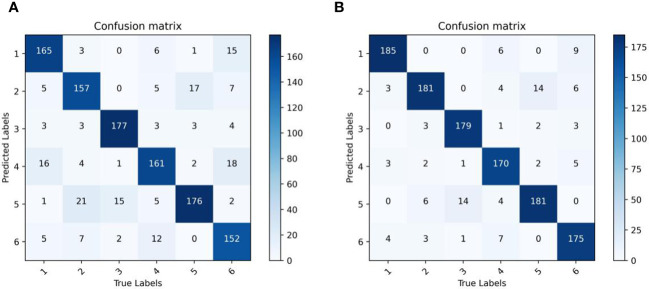
Model confusion matrix visualization. **(A)** Original model confusion matrix visualization. **(B)** Our model confusion matrix visualization.

In order to further validate the recognition capability of the original identification model proposed in this paper, we have provided visual confusion matrix comparison charts for the model before and after improvement in **Figure 15**. It can be observed that the improved network model has effectively reduced the error rates for each category, especially significantly decreasing the misclassification of the first category seed as the fourth category, the misclassification of the second category seed as the fifth category, and the misclassification of the sixth category seed as the fourth category.

In summary, the improved model can better extract fine-grained features such as color and texture information from corn seeds, leading to a significant reduction in recognition error rates. However, the model still needs to improve in identifying seeds in the fourth and fifth categories. Therefore, improving the recognition rates for these particular categories will be a focal point of our future research efforts.

### 3.5 Comparison of related studies

Detailed comparisons with related studies were not feasible in this experiment due to the different methods, datasets, and classification criteria employed. Nonetheless, we compared some applications in agricultural classification tasks, considering several criteria such as dataset size, applications, methods used, and accuracy. The comparisons, as shown in **Table 8**, indicate that the accuracy of different classification tasks is above 85%, with most methods utilizing deep learning models combined with HSI or employing transfer learning. In contrast, the method proposed in this paper achieved an accuracy of over 90% solely using CNN. This demonstrates the rationality of the sample size selection and the effectiveness of the proposed approach. In this scenario, the credibility of this study has been enhanced, providing a valuable reference for agricultural product classification.

**Table 8:** Comparison of the proposed model and related studies (seeds).

Imaging Method	Dataset Size	Application	Approach	Result	References
Digital camera	7500	Variety identification	CNN	85.6%	(Rybacki et al., 2023)
Digital camera	3000	Variety identification	CNNs (transfer learning)	Over 90%	(Altuntaş et al., 2019)
Near-infrared spectroscopy	5400	Variety identification	NIR-HSI+LR/SVM/ CNN/RNN/LSTM	Over 90%	(Zhang et al., 2020)
Near-infrared spectroscopy	1200	Viability prediction	NIR-HSI+CNN	90%	(Ma et al., 2020)
Hyperspectral imaging	3200	Variety identification	HSI+DCNN	93.3%	(Zhang et al., 2021)
Digital camera	5877	Variety identification	Our model	91.23%	Our work

### 3.6 Validation of model generalization ability

To further validate the generalizability and robustness of the model, this study selected the maize dataset used by Chunguang Bi et al (Wang and Song, 2023). The dataset consists of 19 categories of maize seeds, making it representative and challenging. As shown in **Table 9**, the improved model achieved an accuracy increase from 90.17% to 93.96% on this dataset. The analysis of other performance metrics, including precision, recall, and F1 score, also showed significant improvements. This indicates that the improved model can adapt to different maize seed conditions and maintain high performance when faced with new datasets. It demonstrates the effectiveness of the latest model in handling data from various sources and characteristics, highlighting its strong generalization ability and robustness.

**Table 9:** Comparison of the model before and after improvement on a new dataset.

Num	Model	Accuracy	Precisioin	Recall	F1-score
1	ResNet50	90.17%	90.04%	89.91%	0.9002
2	Our Model	93.96%	93.57%	93.53%	0.9355

## 4 Conclusions

Our research involves image acquisition of six different types of corn seeds, namely JD407, JD50, JD83, JD953, JD209, and JD626. We introduce the ResStage structure early in the model to facilitate better information propagation throughout the network layers, thereby promoting the learning process and reducing information loss. In addition, we have introduced both the ECA module and depthwise separable convolution on the residual blocks in the later stages of our model. This simultaneous integration allows us to capture global correlations between features better while significantly reducing the required number of model parameters and computational workload. Finally, we globally introduced the Swish-PReLU hybrid activation function, which combines the unbounded lower-bound, smooth, and non-monotonic properties of the Swish activation function with the adaptive parameter learning capabilities of the PReLU activation function. This was done to enhance the model’s predictive ability for corn seeds. Integrating these three improvements and conducting experiments on datasets comprising six different types of corn seeds demonstrated that the proposed method achieved an impressive accuracy of 91.23%.

Our proposed network model outperforms other commonly used image classification models, including ResNet50, Res2Next50, DenseNet201, ConvNext_T, and RepVgg_A2, in terms of performance while maintaining lower model complexity. Compared to the original network models, our model has achieved a 7.07% increase in accuracy, reduced the loss value by 0.19, decreased the parameter count by 40%, lowered FLOPs by 0.92GMac, and shortened the training time per epoch by 3s.

In conclusion, our proposed method has shown good performance in applying maize seed variety identification. However, seed variety identification involves crucial decisions in agricultural production, such as planting time, fertilization methods, irrigation levels, etc. Moreover, the design and optimization of the model should provide deep insights into seed variety characteristics, growing environmental conditions, and agricultural production management decisions. Therefore, in future research, in addition to considering the impact of factors such as seed storage time, cultivation conditions, and shooting angles on the model’s performance, we will also focus on the model’s management impact and insights into decision-making purposes. This aims to achieve effective support and guidance for seed variety identification and production.

## Data availability statement

The raw data supporting the conclusions of this article will be made available by the authors, without undue reservation.

## Author contributions

JL: Writing – original draft, Writing – review & editing. FX: Writing – original draft, Writing – review & editing. SS: Writing – original draft, Writing – review & editing. JQ: Writing – review & editing.
